# Immediate-Type Respiratory Allergy to Millet-Containing Seed Mixture of Bird Food

**DOI:** 10.1097/WOX.0b013e31817833ef

**Published:** 2008-08-15

**Authors:** Stephanie Rombold, Markus Ollert, Martin Sbornik, Jürgen Rakoski, Ulf Darsow, Johannes Ring

**Affiliations:** 1 Department of Dermatology and Allergy Biederstein, Technische Universität München, Munich, Germany

**Keywords:** bronchial asthma, type I allergy, millet, bird food

## Abstract

A 21-year-old patient experienced asthmatic attacks when cleaning the cage of his budgerigar. Skin tests and radioallergosorbent test were positive to grass pollen and negative for budgerigar feathers and feces. When the food of the bird, a mixture of grain, was tested, a positive reaction to millet was found. Nasal provocation test with millet was positive. Specific immunoglobulin E antibodies against millet were detected in the radioallergosorbent test and in immunoblot analysis. The immunoblot showed specific immunoglobulin E antibodies against a 60-kd protein in millet of birdseed and against a 60-and 36-kd protein in common millet. Immediate-type allergy to millet is rare and occurs mostly as anaphylactic reaction after ingestion of millet but may also occur as asthmatic attack after inhalation of millet.

## 

Allergy to millet is rare. Type I sensitization to millet can lead to asthma by inhalation and to anaphylaxis by ingestion of millet-containing food [1]. A sensitization to millet via inhalation in bird keepers may also elicit food allergy [2]. Millet (*Panicum miliaceum*, Figure 1) belongs to the family of grasses (Poaceae) like barley, oat, rice, or wheat. Cross-reactions between different kinds of grains are possible [3].

**Figure 1:**
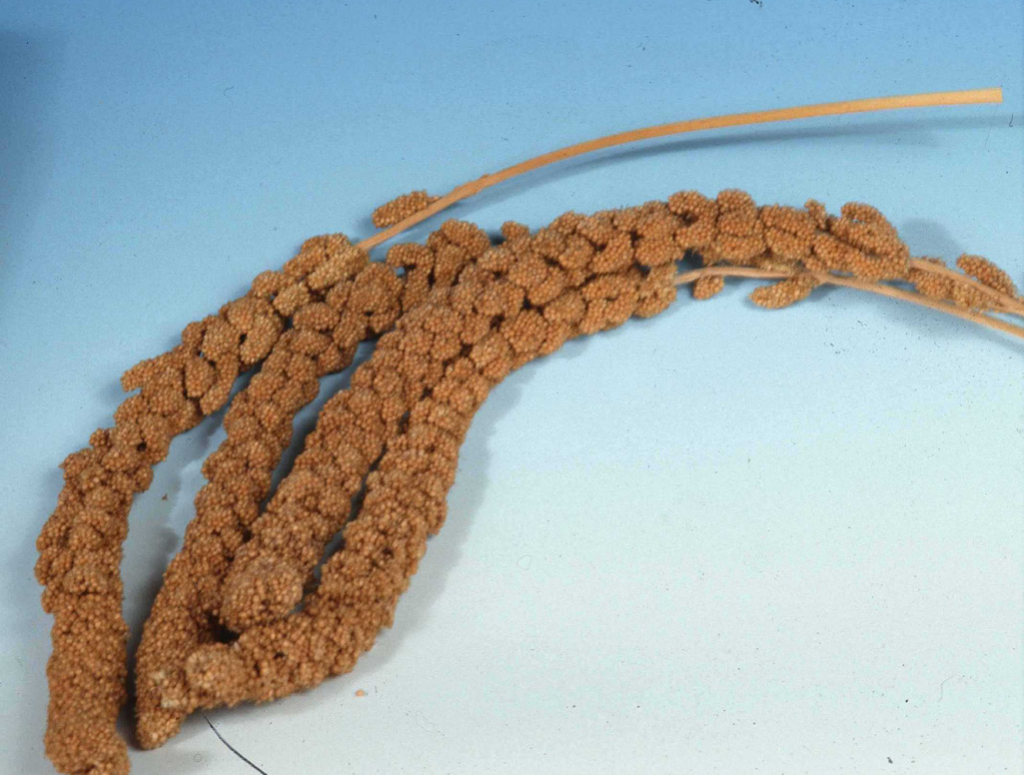
**Common millet (*P. miliaceum*)**.

## Case report

### History

A 21-year-old man experiences perennial allergic rhinoconjunctivitis and bronchial asthma since childhood. Sensitizations to house-dust mite, grass pollen, and cat dander are known. Since 9 months, the patient reported deterioration of asthma and asthmatic attacks when cleaning the cage of his budgerigar. The asthma was well adjusted. The lung function showed no pathological findings. The vital capacity, the forced expiratory volume, and the peak expiratory flow were 6.98 L, 5.33 L, and 12.37 L/s, respectively, and within the reference range. His asthma medications consisted of fluticasone-17-propionate inhalation 2-0-2 and fenoterol inhalation when needed. The family history was positive; his sister was suffering from hay fever.

### Skin prick test

**Figure 2:**
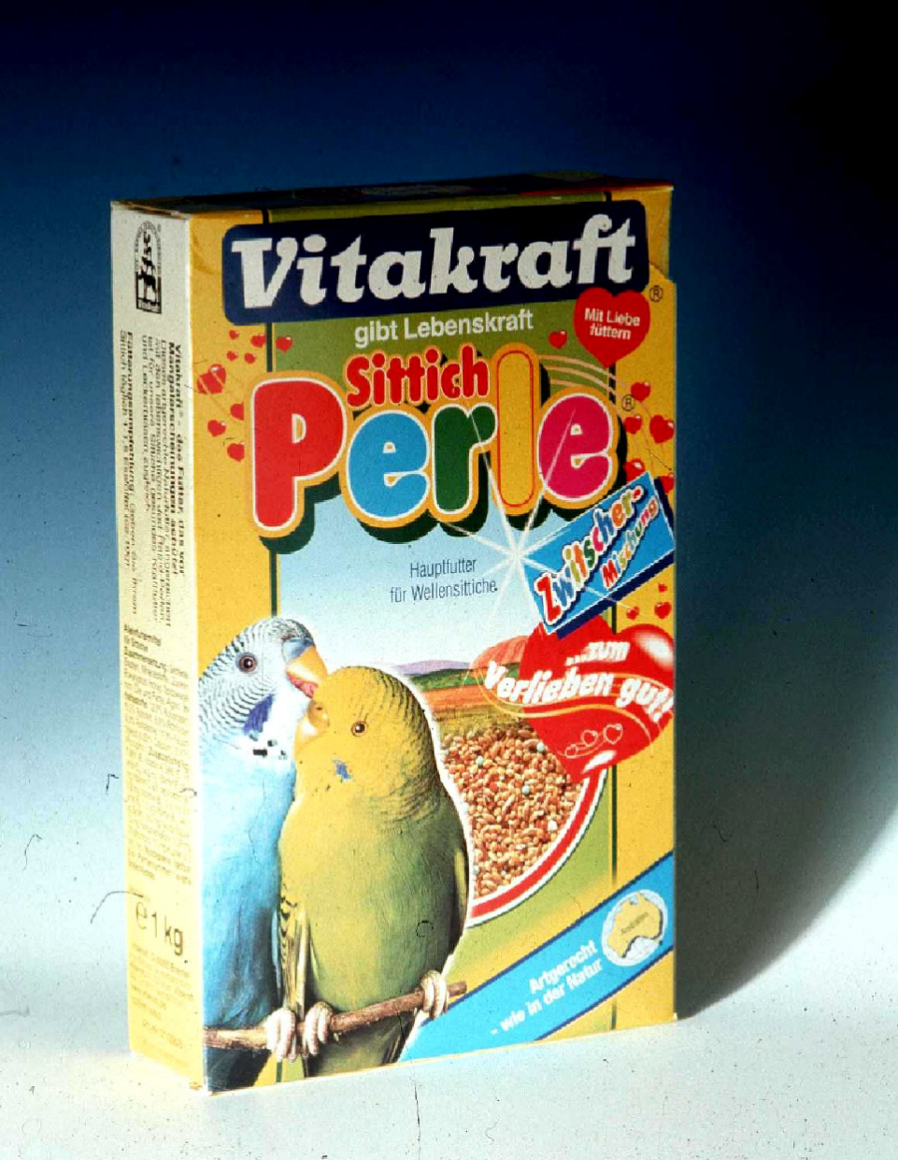
**The package of Budgerigar Twitter Mixture (*Sittich-Perle-Zwitschermischung; Vitakraft*)**.

**Figure 3:**
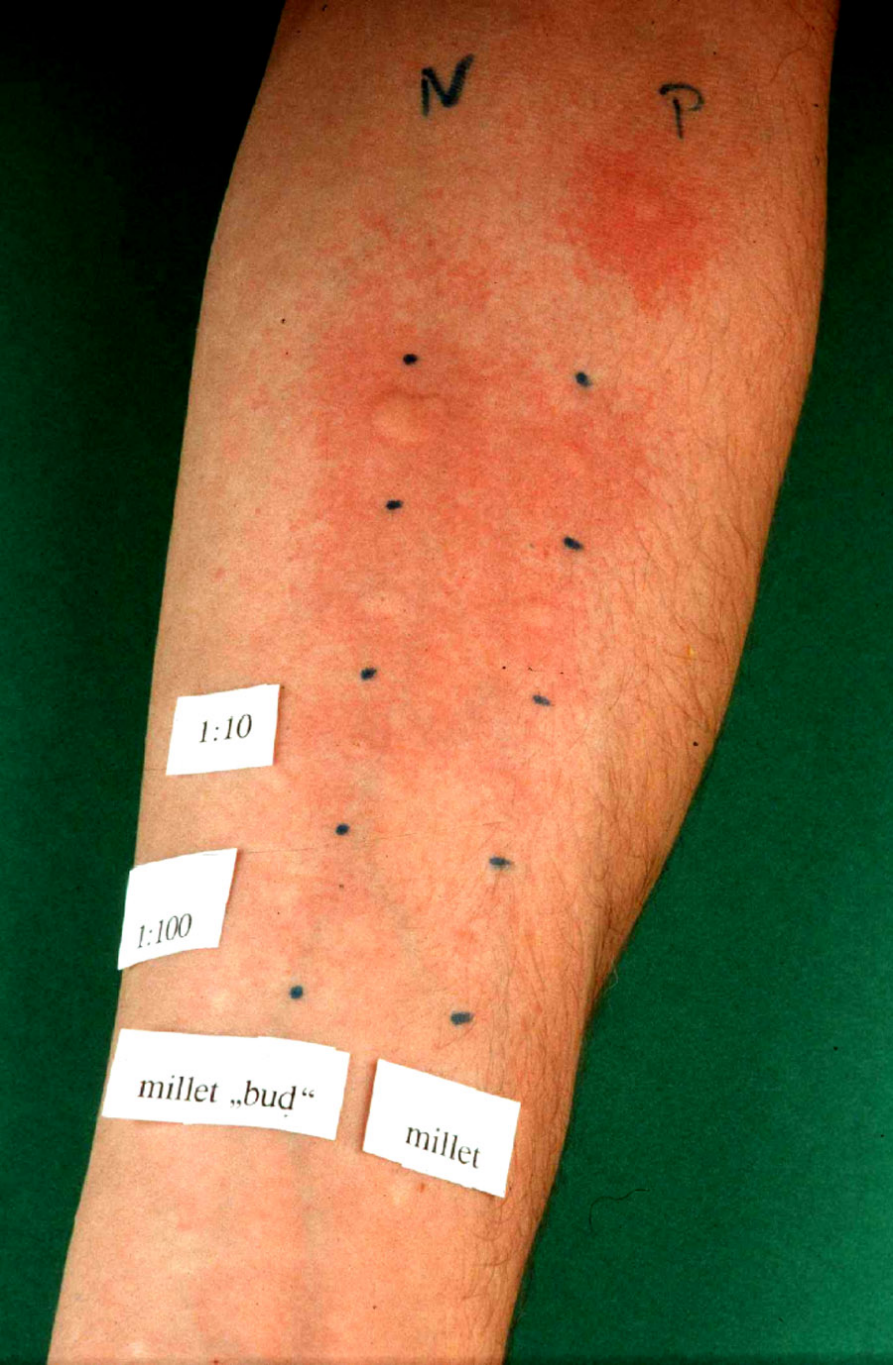
**Positive reaction in the skin prick test to a diluted and undiluted solution: on the left side, the blue component of the birdseed; on the right side, common millet**.

Strongly positive reactions in the skin prick tests[4] (Allergopharma, Reinbek, Germany) were found to house-dust mites, grass pollen, cat dander, and the bird's food, called Budgerigar Twitter Mixture (*Sittich Perle-Zwitschermischung*; Vitakraft, Bremen, Germany; Figure 2). This mixture of grains contains about 8 different seeds, which were all single tested. There were no positive reactions in the skin prick test but to 1 blue component of the seed mixture, which contains among others, vitamins, minerals, fat, and millet. The blue component was tested positive undiluted and in a dilution of 1:10 and 1:100 in saline. To be sure that the positive reaction to the blue seed was to the millet and not to the other ingredients, we then also tested common millet, which yielded a positive result (Figure 3). Weakly positive reactions were found to dog and rabbit dander. The skin prick tests were negative to standard foods, storage mites, the bird's feathers, sand, and feces.

### In vitro test

**Figure 4:**
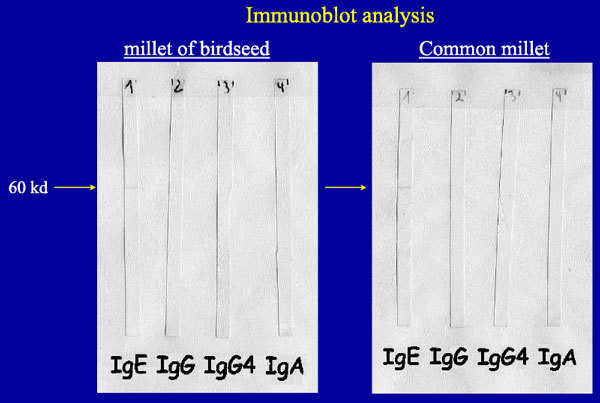
**Specific IgE antibodies against a 60-kd protein in millet of birdseed (left) and against a 60-kd protein in common millet (right) in the immunoblot analysis**.

Further allergy tests were done to confirm our diagnosis of immediate-type allergy to millet in bird food. We found specific immunoglobulin E (IgE) antibodies[5] against common millet in the CAP radioallergosorbent test (RAST) fluoroenzyme immunoassay with 12.8 kU/L (class 3) and specific IgE antibodies against a 60-kd protein in millet of birdseed, and against a 60-and 36-kd protein in common millet in the immunoblot analysis[5-8] (Figure 4). The RAST inhibition assay[9] with *Phleum pratense*, which was done to study cross-reactivity with IgE antibodies between grass pollen and millet, showed no inhibition.

### Provocation test

The nasal provocation test[10,11] with millet was positive.

### Recommendation

Because millet is in most of the bird's food, it is difficult to dispense with millet. So the patient avoided cleaning the cage of his budgerigar, and the asthma attacks stopped.

## Discussion

Asthma and rhinoconjunctivitis after inhalation of millet in bird food are rare. So far, 1 case in the literature is described by Stuck et al,[12] where cleaning of the birdcage led to asthma attacks. In this case, the patient also experienced anaphylaxis after ingestion of millet. The sensitization to millet via inhalation of millet in birdseed may later lead to manifest food allergy [2]. Anaphylactic reactions after ingestion of millet are more common. There are about 10 cases described since 1981 [2,12-14]. Anaphylactic reactions after ingestion of millet in our patient are not known in contrast to the study described by Stuck et al.[12]

Cross-reactivity between cereal grains in the Poaceae family is not uncommon. A 16-kd rice protein is supposed to be one of the major allergens in rice grain extract and may be responsible for cross-allergenicity [3]. Because the RAST inhibition assay in our patient between grass pollen and millet showed no decrease in IgE binding, the asthma attacks in our patient were caused by sensitization to millet and not due to cross-reactivity to grass pollen allergens as the basis of this hypersensitivity.

In conclusion, not only bird's feathers but also seeds in bird food can represent an additional risk factor for atopic bird keepers.
